# APGW/AKH Precursor from Rotifer *Brachionus plicatilis* and the DNA Loss Model Explain Evolutionary Trends of the Neuropeptide LWamide, APGWamide, RPCH, AKH, ACP, CRZ, and GnRH Families

**DOI:** 10.1007/s00239-023-10146-9

**Published:** 2023-12-16

**Authors:** Cristian E. Cadena-Caballero, Nestor Munive-Argüelles, Lina M. Vera-Cala, Carlos Barrios-Hernandez, Ruben O. Duarte-Bernal, Viviana L. Ayus-Ortiz, Luis A. Pardo-Díaz, Mayra Agudelo-Rodríguez, Lola X. Bautista-Rozo, Laura R. Jimenez-Gutierrez, Francisco Martinez-Perez

**Affiliations:** 1 Grupo de Investigación Computo Avanzado y a Gran Escala (CAGE), Escuela de Ingeniería de Sistemas e Informática, Universidad Industrial de Santander, 680002 Bucaramanga, Colombia; 2 Biomedical Imaging, Vision and Learning Laboratory (BIVL2ab), Escuela de Ingeniería de Sistemas e Informática, Universidad Industrial de Santander, 680002 Bucaramanga, Colombia; 3 Grupo de Investigación en Demografía, Salud Pública y Sistemas de Salud (GUINDESS), Departamento de Salud Pública, Universidad Industrial de Santander, 680002 Bucaramanga, Colombia; 4 Facultad de Ciencias del Mar, Universidad Autónoma de Sinaloa, 82000 Mazatlán, México; 5 Cátedra-CONAHCyT, Consejo Nacional de Humanidades Ciencias y Tecnología, 03940 CDMX, México; 6 Laboratorio de Genómica Celular Aplicada (LGCA), Grupo de Microbiología y Genética, Escuela de Biología, Universidad Industrial de Santander, 680002 Bucaramanga, Colombia

**Keywords:** APGWamide, Adipokinetic hormone family, Gonadotropin-releasing hormone family, Invertebrate neuropeptides, Evolution, DNA loss model

## Abstract

In the year 2002, DNA loss model (DNA-LM) postulated that neuropeptide genes to emerged through codons loss via the repair of damaged DNA from ancestral gene namely *Neuropeptide Precursor Predictive* (*NPP*), which organization correspond two or more neuropeptides precursors evolutive related. The DNA-LM was elaborated according to amino acids homology among LWamide, APGWamide, red pigment-concentrating hormone (RPCH), adipokinetic hormones (AKHs) and in silico APGW/RPCH *NPP*APGW/AKH *NPP* were proposed. With the above principle, it was proposed the evolution of corazonin (CRZ), gonadotropin-releasing hormone (GnRH), AKH, and AKH/CRZ (ACP), but any *NPP* never was considered. However, the evolutive relation via DNA-LM among these neuropeptides precursors not has been established yet. Therefore, the transcriptomes from crabs *Callinectes toxotes* and *Callinectes arcuatus* were used to characterized ACP and partial CRZ precursors, respectively. BLAST alignment with APGW/RPCH *NPP* and APGW/AKH *NPP* allow identified similar *NPP* in the rotifer *Brachionus plicatilis* and other invertebrates. Moreover, three bioinformatics algorithms and manual verification were used to purify 13,778 sequences, generating a database with 719 neuropeptide precursors. Phylogenetic trees with the DNA-LM parameters showed that some ACP, CRZ, AKH2 and two *NPP* share nodes with GnRH from vertebrates and some of this neuropeptide had nodes in invertebrates. Whereas the phylogenetic tree with standard parameters do not showed previous node pattern. Robinson-Foulds metric corroborates the differences among phylogenetic trees. Homology relationship showed four putative orthogroups; AKH4, CRZ, and protostomes GnRH had individual group. This is the first demonstration of *NPP* in species and would explain the evolution neuropeptide families by the DNA-LM.

## Introduction

Neuropeptides participate in a wide variety of autocrine, paracrine, endocrine, and neuroendocrine communication mechanisms (Merighi 2009; Burbach 2011). These peptides are synthesized from a precursor (pre-pro-peptide) that contains a signal peptide from the rough endoplasmic reticulum, one or more active neuropeptides, the related peptides, and an excision motif for endoproteases that recognize a few different combinations of basic amino acids depending on the neuropeptide precursor (Rouillé et al. 1995; Hökfelt et al. 2000).

Based on the variations in the amino acid sequence of the active peptide and the structure of its precursor, some neuropeptides have been grouped into distinct families (Hoyle 1998). In invertebrates, these neuropeptide families include LWamide and APGWamide, which are grouped according to their structural similarities but have more than one active peptide that differs at the C-terminus (Nässel and Taghert 2006). Particularly, leucine is absent in APGWamide, whereas alanine is replaced by another amino acid residue (Martínez-Pérez et al. 2002, 2007).

Other neuropeptides that contain homologous amino acids at the carboxylic end of LWamide and APGWamide are named according to their physiological activity, including the red pigment-concentrating hormone (RPCH) and adipokinetic hormone (AKH) (Josefsson 1983). Both neuropeptides occur in monocopy, and their precursor consists of eight amino acids. However, some AKHs can have nine to twelve amino acids (Martínez-Pérez et al. 2007). Additionally, a phenylalanine residue is typically present in the fourth position from the N-terminal of both neuropeptides (Josefsson 1983; Gäde 2009; Gäde et al. 2020). Another similarity between the members of the RPCH and AKH families is that they have a tryptophan residue and an amidated glycine in the C-terminus (Martínez-Pérez et al. 2007). This tryptophan of the RPCH plays a critical role in the aggregation of intracellular pigments of chromatophore cells in the Baltic prawn *Leander adspersus* (Christensen et al. 1978), as well as in the AKH from desert locust *Schistocerca gregaria* (Christensen et al. 1979). Eleven years later, the APGWamide neuropeptide from the sea snail *Fusinus ferrugineus* was purified (Kuroki et al. 1990). It is also worth noting that the neuropeptide contained the carboxyl-terminal amino acid sequence that was synthesized, to demonstrate the indispensable of this region to the physiological activity of RPCH and AKH (Christensen et al. 1978). The following year, the physiological role of tryptophan and amidated glycine of APGWamide was confirmed in several types of mollusks (Minakata et al. 1991).

To determine the evolutionary relationship between these neuropeptide families, the DNA loss model (DNA-LM) was proposed at two different points. In the first instance (Martínez-Pérez et al. 2002), the model was supported by the studies of the amino acid activity in RPCH and AKH obtained from Christensen et al. (1978, 1979) and with the APGWamide reported by Kuroki et (1990). The codons of APGWamide precursors of the great pond snail *Lymnaea stagnalis*, blue mussel *Mytilus edulis,* and California sea hare *Aplysia californica* were aligned to the RPCH of two crabs and the AKH of five insects. The results demonstrated that the codons for the first four amino acids of RPCH and AKH were located in two regions between the first and second copy of the molluscan APGWamide precursor and the next four codons of amino acids essential for the activity of all neuropeptides were common in the third copy of APGWamide. RPCH/AKH was generated from the merging of homologous codons from these molluscan species and their translation, which was referred to as a virtual peptide (Martínez-Pérez et al. 2002). Furthermore, the presence of two other homologous regions in the codons of APGWamide precursors with respect to the position of the introns in RPCH and AKHs precursors genes was determined. The DNA-LM proposed that there should be an intron in the APGWamide precursor gene in these regions, which would be a reflection of the possible ancestral gene (Martínez-Pérez et al. 2002).

In the second instance, the union of all the conserved domains of codons was analyzed in the APGWamide precursors of the three mollusks with respect to RPCH and AKH (Martínez-Pérez et al. 2007). These generated precursors containing one to three copies of APGWamide and one copy of RPCH/AKH were termed RPCH/AKH virtual precursors, hereinafter referred to as APGW/RPCH Neuropeptide Precursor Predictive* (NPP)* and APGW/AKH *NPP*. Interestingly, these *NPP* showed homology with the *Hydra* copies of the LWamide precursor, with the only difference being that the LWamide leucine was neither in the APGWamide nor in the last four codons of RPCH and AKH (Martínez-Pérez et al. 2007). Therefore, the model proposes that the evolution of neuropeptide genes occurred through the duplication of an ancestral gene, where one paralog contained codons of LWamide that coded for neuropeptide precursors with different copy numbers. Therefore, new domains were generated in phylogenetically related species due to nucleotide fission, loss of codons, movement of introns, and fusion of conserved codons generating LW/APGW *NPP* (Martínez-Pérez et al. 2002, 2007).

Given the postulation of the DNA-LM, several studies have sought to identify genes and/or mRNA for *NPPs* in invertebrates, under the expectation that *NPPs* could be identified in genomes from species whose body plan was similar to species from the Cambrian explosion (Yue et al. 2014; Li and Ni 2016). However, no conclusive data could be obtained due to the limitations of the experimental methods of the time. In 2010, with the introduction of next-generation sequencing (NGS) technologies, the genomic sequences of marine invertebrates such as* A. californica* (Fiedler et al. 2010) were obtained, thus confirming the intron positions proposed in the DNA-LM for the evolution of the APGWamide precursor (unpublished data). In later studies, an AKH-like protein was characterized in *A. californica* (Johnson et al. 2014). With the widespread adoption of NGS, similar genes previously proposed by the DNA-LM were reported in invertebrates, including the AKH/Corazonin-related peptide (ACP) (Hansen et al. 2010). Interestingly, corazonin (CRZ) was characterized in the American cockroach in 1989 and no similar neuropeptide to AKH had been reported at that time (Veenstra 1989). This also occurred with gonadotropin-releasing hormone (GnRH) from mammals (Morgan and Millar 2004), which was isolated and characterized from brains from the common octopus *Octopus vulgaris* (Iwakoshi et al. 2002). Later on, with the cloning of its neuropeptide precursor, the homology with CRZ was established and it was classified as GnRH-like (Hauser and Grimmelikhuijzen 2014), but it has been proposed that it corresponds to the members of the CRZ family (Tsai 2018; Zandawala et al. 2018).

Collectively, the aforementioned findings led to the postulation of various evolutionary models to explain the evolution of these neuropeptide families primarily based on the sequences of the active peptides or the peptide precursor (Hauser and Grimmelikhuijzen 2014; Roch et al. 2014; Plachetzki et al. 2016; Tian et al. 2016; Sakai et al. 2017; Tsai 2018; Zandawala et al. 2018). Nevertheless, no previous studies had considered the principles of codon loss in LWamide and APGWamide precursors that could generate the APGW/RPCH *NPP* or APGW/AKH *NPP*, as proposed by the DNA-LM (Martínez-Pérez et al. 2002, 2007) and used to explain neuropeptides evolution and diversity in Metazoan (De Oliveira et al. 2019; Jékely 2013).

This study provides an in silico demonstration that the genome of the rotifer *Brachionus plicatilis* (Blommaert et al. 2019) contains a gene without introns that codes for a precursor with three copies of APGWamide and one AKH, which is homologous to two of the proposed APGW/AKH *NPPs* by the DNA loss model according to studies conducted in 2002 and 2007 (Martínez-Pérez et al. 2002, 2007).

Moreover, the homologous genes to the precursors of the APGWamide, AKH, CRZ, cerebral peptide, prothoracicostatic peptide (PTSP), and neuropeptide precursors from genomes reported in the GenBank database with hypothetical nomenclature were analyzed too. Additionally, with NGS analyses, the precursors of ACP from *Callinectes arcuatus* and CRZ from *Callinectes toxotes* were characterized. The evolutionary pathways of these neuropeptide families and their implications in the function of the DNA loss model are discussed below.

## Materials and Methods

### Transcriptomes from *Callinectes arcuatus *and *Callinectes toxotes*

As previously reported by Jimenez-Gutierrez et al. (2019), *Callinectes arcuatus* and *Callinectes toxotes* were captured on board a fishing boat in the Sea of Cortez, Pacific Ocean (23°20′N 106°30′W). The ovaries, hepatopancreas, and eyestalks of the captured organisms were removed and stored at − 80 °C without buffer or cell culture medium. The samples from 10 females and 2 males in total were then processed to obtain transcriptomes that were representative of all stages of maturity, the different capture seasons, and the circadian rhythm for each species. The total RNA was first extracted using the Pure Link RNA Mini Kit (Invitrogen/Thermo Fisher Scientific, Waltham, MA) and a second extraction was conducted by using a modified version of the TRIzol method in which the centrifugation times and total RNA precipitation protocols were optimized. Library construction was conducted according to the TruSeq Stranded mRNA preparation guide (Illumina, San Diego, CA) and the products were sequenced with an Illumina MiSeq sequencer (RRID:SCR_016379) according to the manufacturer’s instructions.

As reported by Jimenez-Gutierrez et al. (2019), adapter sequences were eliminated from all of the raw reads and low-quality reads were discarded using the Trim-galore software (RRID: SCR_011847). Normalization was conducted using Trinity version 2.6.6 (RRID:SCR_013048). De novo assembly was conducted using SPAdes version 3.12.0. (RRID:SCR_000131). To establish the relationship between the assembled sequences and their function, a BLAST alignment (RRID:SCR_004870) was conducted using Diamond version 0.9.22 (RRID:SCR_016071) against the “nucleotide collection” database from the National Center for Biotechnology Information (NCBI, RRID:SCR_006472). The putative neuropeptide precursor sequences corresponding to the families examined were verified via BLAST with the GenBank databases (RRID:SCR_002760).

### Identification of Neuropeptide Precursor Families in the GenBank Database

The identification of all potential amino acid sequences of the neuropeptide precursors from all species was conducted using the GenBank database with keywords related to each neuropeptide precursor until April 8th, 2020, whereas the members of the ACP family were identified with BLAST using the GenBank database (Boratyn et al. 2013; Sayers et al. 2019). Only the identified sequences of the AKH family were subdivided as previously reported (Vroemen et al. 1998) (see Repository 1 for more details). The neuropeptide precursors were identified using three bioinformatic algorithms coupled with manual curation based on the presence of proteolytic cutting sites for the post-translational processing of the neuropeptide precursors.

BioDataToolKit version 5 software (https://github.com/rduarte24/BiodataToolkit) was used to automatically download the neuropeptide sequences from the GenBank database (Sayers et al. 2019) and to eliminate the sequences that did not contain a neuropeptide structural organization. Then, sequences that presented dibasic amino acids for the proteolytic cleavage were identified with Proteios version 1.0 (https://github.com/Martin-Munive/Proteios). Finally, the selected precursors with potential sites for pro-protein convertase were determined with ProP 1.0 Server (RRID:SCR_014936) (Duckert et al. 2004).

The results and the remaining combinations of basic amino acid pairs were verified manually. After obtaining, purging, and verifying the neuropeptide precursor sequences, a database was created with a detailed description of each of the selected neuropeptide precursor sequences (see Repository 1 for more details).

### In silico Validation of Predicted Neuropeptides Precursors from the DNA-LM

BLAST protein–protein alignments with respect to the NCBI database were performed with the APGW/RPCH *NPP* and APGW/AKH *NPP* proposed by the DNA-LM (Martínez-Pérez et al. 2007; Boratyn et al. 2013). Non-redundant protein sequence database searches were conducted without excluding organisms, models, non-redundant RefSeq proteins, or uncultured/environmental sample sequences. The general parameters were the following: maximum number of aligned sequences, 20,000; expected number of chance matches in a random pattern, 100; length of the seed that starts an alignment, 6; and limit of the number of matches to a query range, 0; word size was automatically adjusted to improve the results for short queries. For the scoring parameters, the BLOSUM62 matrix was used with conditional compositional scoring matrix adjustment and gap cost of existence: 11; extension: 1. Finally, for the filters and masking, the “low complexity regions,” “mask lower case letters,” and “mask for lookup table only” options were not used (Boratyn et al. 2013). The correlation between the APGW/RPCH *NPP* and APGW/AKH *NPP* sequences with the homologous precursors selected from the BLAST result was edited with GeneDoc version 2.7 (Nicholas 1997).

The alignment for each one of the neuropeptide precursor families was performed using the Kalign software version 2.0 (RRID:SCR_011810) (Lassmann and Sonnhammer 2005; Lassmann et al. 2009) from the European Bioinformatics Institute (EMBL-EBI; RRID:SCR_004727) (Madeira et al. 2019), with the default parameters and with the DNA-LM parameters (Martínez-Pérez et al. 2007): gap open penalty, 9; gap extension penalty, 0.2; terminal gap penalties, 0.45; and bonus score, 0.0.

### Phylogenetic Relationship Among *NPP* Families

Phylogenetic trees from each neuropeptide precursor were generated using the IQ-tree software version 1.6.12. (RRID:SCR_017254) (Nguyen et al. 2015). To this end, the number of CPU cores used in each run was automatically established with the *-nt AUTO* command and the substitution models were determined with ModelFinder (Kalyaanamoorthy et al. 2017). Branch support analysis was obtained with *-bb 1000* for the bootstrap ultrafast method (Minh et al. 2013; Hoang et al. 2018). To achieve this, the partition type used was *Edge-linked* with *FreeRate* (+ *R*) heterogeneity in four categories and empirical frequency status. The type of amino acid sequences was specified with *-st AA*. Likewise, the number of initial parsimony trees was determined with *-ninit 100* and the number of trees to maintain during software execution was established with *-nbest 5*. The single branch test with 1000 replicates (SH-aLRT) and the approximate Bayes test (aBayes) were conducted, with a minimum correlation coefficient of 0.99 (Guindon et al. 2010; Anisimova et al. 2011). Extended model selection was performed with the *-m TESTNEW* command and Jackknife support was added with *-j 0.3*. Similarly, a disturbance strength of 0.5 and an IQ-Tree stopping rule of 100 were used.

The phylogenetic trees from all neuropeptide precursors groups were modeled in Mesquite version 3.61 (RRID:SCR_017994) (Maddison and Maddison 2019) based on the alignments generated by Kalign version 2.0, and their amino acid substitution models were also evaluated with ModelFinder (Kalyaanamoorthy et al. 2017). The final assembly was run with IQ-tree on the GUANE-1 supercomputer of the Industrial University of Santander (http://wiki.sc3.uis.edu.co/index.php/Cluster_Guane). Finally, the trees were visualized with iTOL version 4.0 (RRID:SCR_018174) (Letunic and Bork 2019).

The metric comparison of the structure between the trees generated with both parameters was performed with Robinson-Foulds metric that quantified the differences and compared the phylogenetic trees according to their distances between branches and positions of taxa used. This was done using IQ-tree software (Robinson and Foulds 1981; Nguyen et al. 2015). In addition, homology relationships between neuropeptide families were established by generating orthogroups that correspond to the set of related neuropeptide precursors with OrthoFinder software version 2.5.5 (RRID:SCR_017118) (Emms and Kelly 2015, 2019).

## Results

### Neuropeptide Precursor Sequences Obtained by NGS

**Fig. 1:**
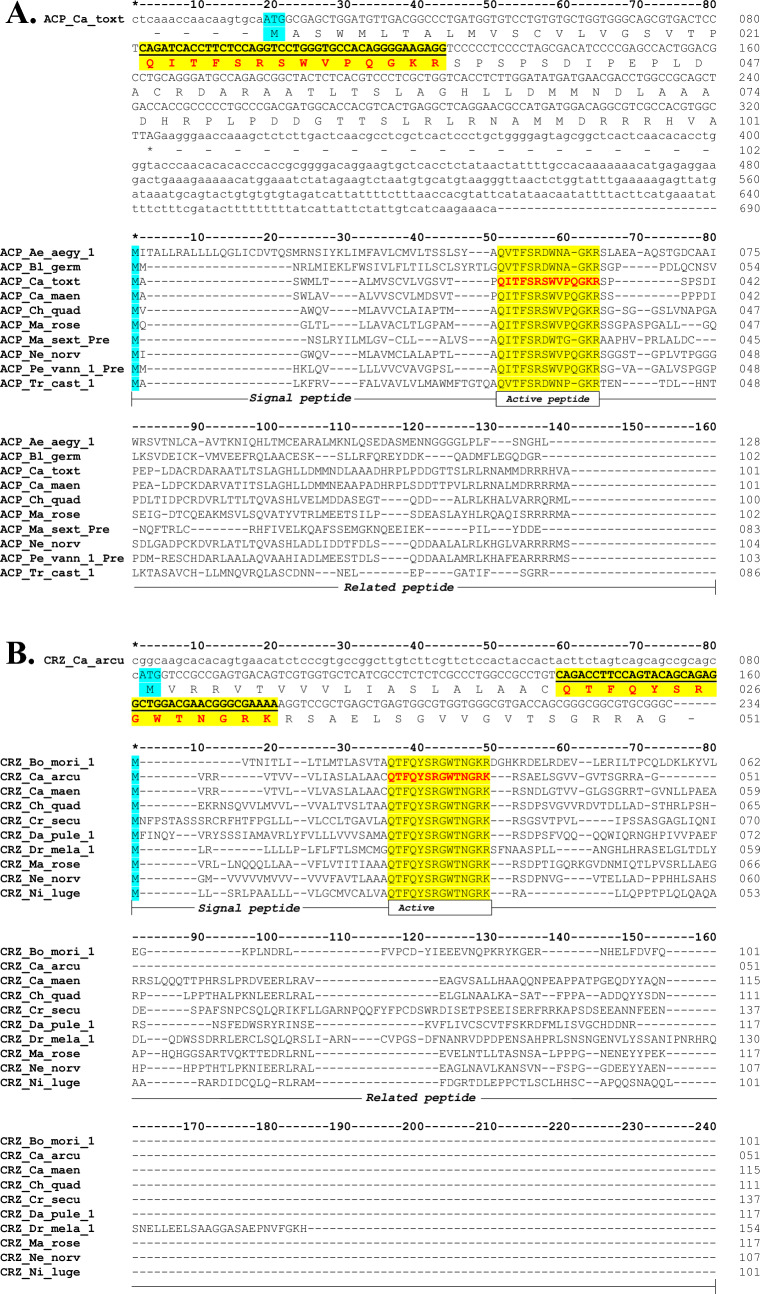
ACP of *Callinectes toxotes* and CRZ of *Callinectes arcuatus*. **A** cDNA sequence of ACP_Ca_toxt (MT488396) and **B** partial sequence of CRZ_Ca_arcu (MT488323) obtained by NSG. The lowercase letters correspond to the 5′ and 3′ UTR sequences. The uppercase letters indicate the ORF. Blue boxes indicate the position of methionine (M). Yellow boxes show the position of the active peptide. The nucleic acids that compose the active peptide are shown in italics and underlined and their conceptual translation is indicated in red font. For both figures, an alignment is included indicating the parts of the precursor, their size, and the total number of amino acids

The complete ACP precursor sequence of *Callinectes toxotes* was characterized via transcriptomic analysis. This sequence exhibited an open reading frame (ORF) of 306 bp, with a 3′ UTR of 366 bp (Fig. 1A). BLAST analyses indicated that the ACP of *Callinectes toxotes* exclusively aligned with the ACP of other crustacean species: 76% with respect to *Carcinus maenas* ACP and 37–44.6% with the remaining species. In *Callinectes arcuatus*, a partial 234 bp sequence of CRZ corresponding to the 5′ UTR of 81 bp of the C-terminus was detected. However, the stop codon was not identified (Fig. 1B). The CRZ sequence shared similarities with those from Crustacea, Insecta, and Chelicerata and with non-tagged sequences or hypothetic CRZ from the phylum Tardigrada and class Gastropoda (Repository 2).

### In silico Identification of Neuropeptide Precursors

From the GenBank databases, 13,778 potential neuropeptide precursor sequences from both invertebrate and vertebrate species were identified. Among these, APGWamide precursor sequences were the least abundant, followed by LWamide, RPCH, and CRZ, whereas AKHs and GnRH precursors were the most abundant (Repository 3). A total of 2294 potential neuropeptide precursors and other non-related sequences were identified using the BioDataToolKit software version 5. From them, 912 potential neuropeptide precursors with dibasic proteolytic cleavage site were identified using the Proteios version 1.0 software. Using the Prop 1.0 Server and manual curation in some cases, only 636 sequences exhibited the dibasic proteolytic cleavage sites for neuropeptide precursors. Additionally, 45 and 38 precursors for ACP and *NPPs* were identified via BLAST sequence alignments, respectively (Repository 1).

**Fig. 2:**
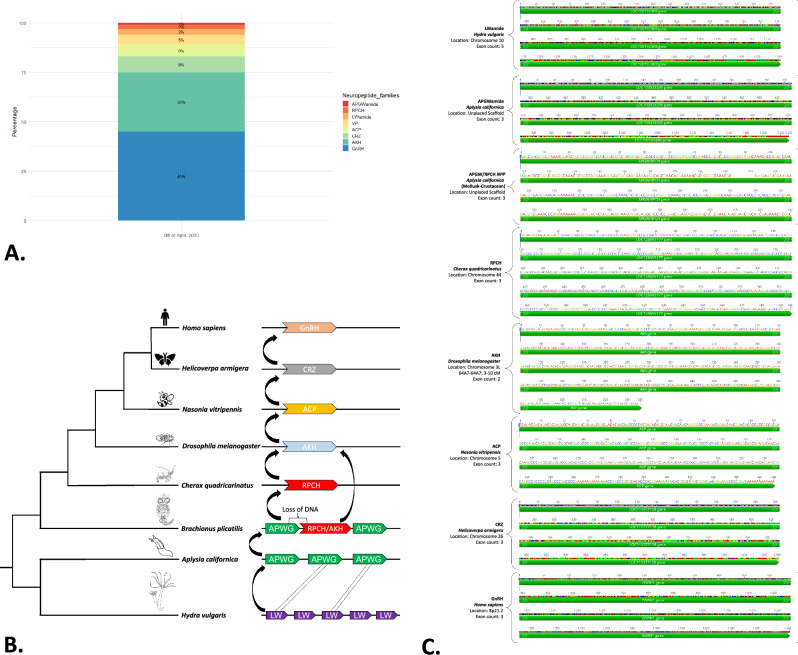
Neuropeptide precursor families. **A** Percentage of sequences for each neuropeptide precursor family obtained from the GenBank database. **B** Schematic of a plausible evolutionary relationship between the neuropeptide families evaluated herein. **C** Example of loci with a model species for each neuropeptide family

From the depurated sequences, a database of 719 neuropeptide precursors was used for evolutionary analysis (Fig. 2). The neuropeptide precursors represented seven neuropeptide families distributed among species from 11 phyla, 30 classes, 85 orders, 181 families, 277 genera, and 368 species. These taxonomic groups included species of fish, insects, mammals, mollusks, and crustaceans (Repository 3). The following observations were made upon inspecting this database: (1) most of the identified neuropeptide precursors corresponded to bioinformatically assembled products; (2) every neuropeptide family, with the exception of RPCH, which is exclusively expressed in crustaceans, exhibited more than one neuropeptide precursor in some phyla; (3) the GnRH family was the most widely represented, occurring in six phyla, whereas some of the remaining families were only present in invertebrate phyla. The aforementioned findings were corroborated by conducting phylogenetic tree analyses for each neuropeptide precursor family (Repository 4).

### BLAST Analysis of the APGW/RPCH and APGW/AKH Neuropeptide Predictive Precursors

**Fig. 3:**
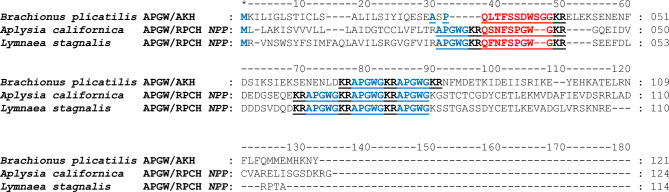
Alignment of the APGW/RPCH *NPP* from *A. californica* and *L. stagnalis* and the APGW/AKH from the rotifer *B. plicatilis*. Similarities between the amino acids of the APGW/RPCH *NPP* of mollusks proposed in 2001 and those of the rotifer *B. plicatilis* (APGW/AKH). The APGWamide copies are indicated in blue and underlined. The RPCH and AKH are indicated in red. The KR proteolytic sites are indicated in bold

**Fig. 4:**
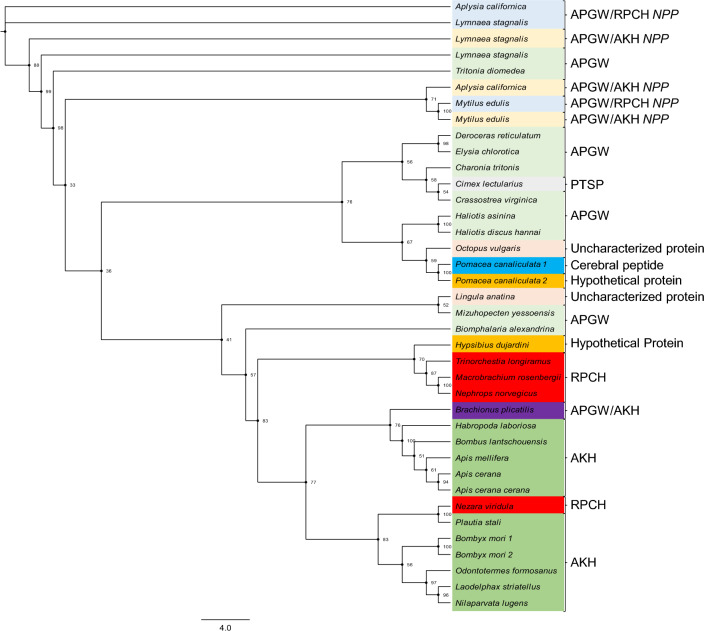
Phylogenetic tree of APGW/RPCH *NPP* and APGW/AKH *NPP*. Phylogenetic relationships between the APGW/RPCH *NPP* and APGW/AKH *NPP* proposed in 2007 and the APGW/AKH precursors reported in the rotifer *B*. *plicatilis* (box-purple) with other species in the GenBank database. RPCH, reported as AKH from *Nezara viridula*, is shown inside the red box (Color figure online)

BLAST analysis of the APGW/RPCH *NPP* and APGW/AKH *NPP* sequences from *A. californica, Lymnaea stagnalis*, and *M. edulis* was homologous to 56 neuropeptide precursors, as predicted by the DNA-LM (Martínez-Pérez et al. 2007). The APGW/AKH *NPP* from *A. californica* and *L. stagnalis* shared similarities with a sequence from the rotifer *B. plicatilis* reported as hypothetic (GenBank code RNA39930.1), which exhibited a decapeptide structure similar to AKHs, as well as two out of the four copies of APGWamide (Fig. 3). Additionally, a neuropeptide precursor from the RPCH family was identified in the hemipteran *Nezara viridula*, which was reported as AKH (Fig. 4).

The APGW/RPCH *NPP* and APGW/AKH *NPP* and the 32 remaining neuropeptides exhibited homology and phylogenetic relatedness with cerebral peptide, AKH, PTSP, hypothetic peptides reported in GenBank, and uncharacterized proteins. The active peptide and the related peptide from the APGW/RPCH *NPP* from *A. californica* exhibited similarities with the neuropeptide precursors from *Macrobrachium rosenbergii* (ANT96502.1). Furthermore, the APGW/AKH *NPPs* from *L. stagnalis* shared similarities with the AKH1 from hemipterans such as *Plautia stali*, *Laodelphax striatellus*, *Nilaparvata lugens*, the tardigrade *Hypsibius dujardini*, and other proteins such as PTSP, APGWamide, and cerebral peptide. On the other hand, APGW/RPCH *NPP* from *M. edulis* was only similar to APGWamide and cerebral peptide (Fig. 4 and Repository 5).

### Constant and Variable Motifs of the Neuropeptide Precursors

**Fig. 5:**
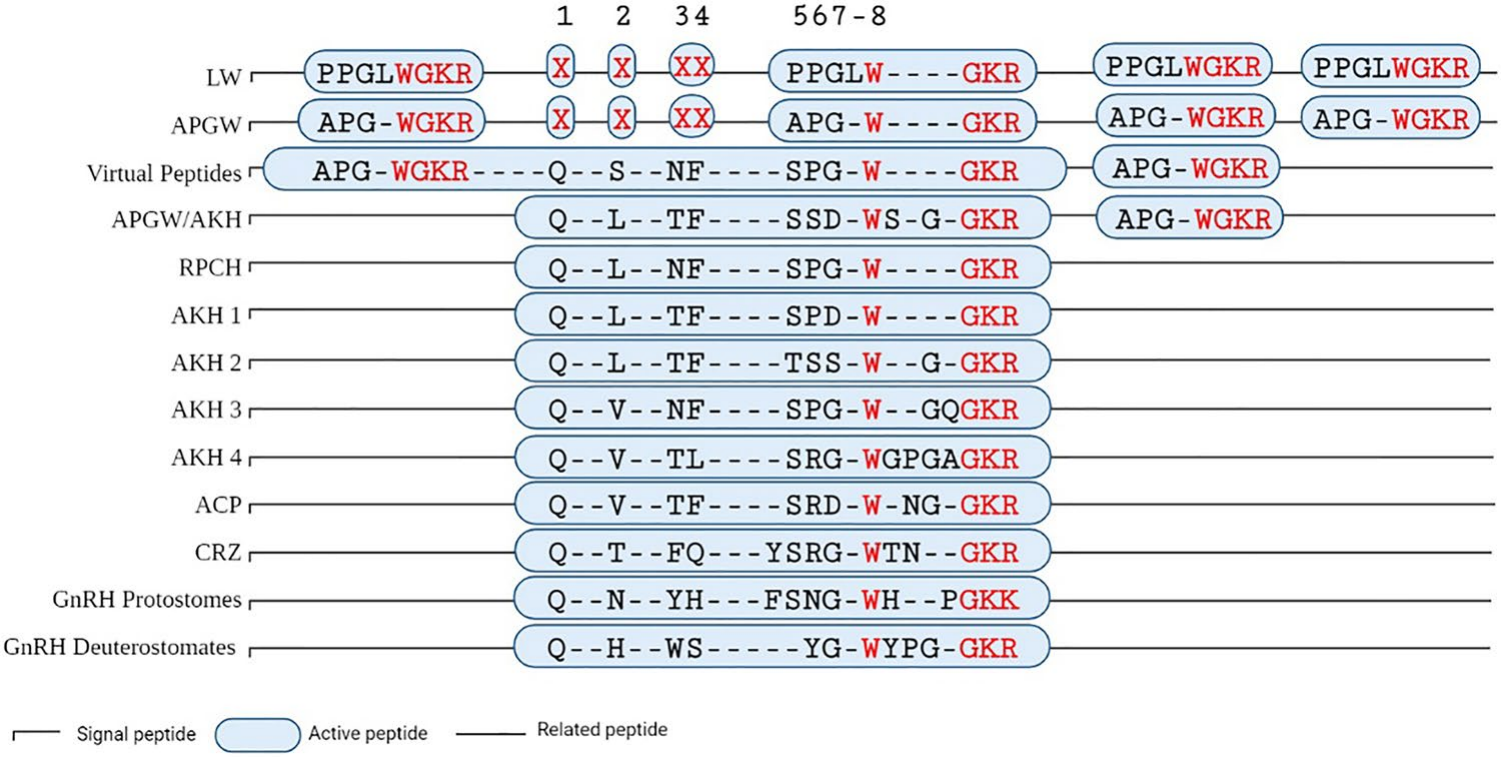
Alignment of active peptides from GnRH family members generated by the DNA-LM. Alignment from LWamide, APGWamide, RPCH, AKHs, CRZ, and GnRH, and APGW/RPCH *NPP* and APGW/AKH *NPP* showing the amino acids within the precursor for LWamide and APGWamide. The blue box indicates the amino acid variations inside the neuropeptide precursors families. The red “X” indicates the absence of amino acids in positions one and four of the related peptide, as well as conserved amino acids. Note that tryptophan position 8, glycine, and the dibasic amino acids were conserved among all families. See Repository 6 for more details on the alignment procedure (Color figure online)

The motifs with the highest variability among the neuropeptide precursors corresponded to the rough endoplasmic reticulum internalization signal peptide, the related peptide, and the Cys region from the C-terminus (Repository 6). In contrast, the most conserved motifs were present in the active peptide and the excision motifs. Progressive alignments of the neuropeptide precursors among families indicated homology between active peptides from LWamide and APGWamide. However, the leucine that characterizes the former family was absent in the latter. The three first amino acids and the phenylalanine from RPCH and AKH1 were also found in two copies from LWamide and APGWamide. Furthermore, the last four amino acids from one of the copies of LWamide and APGWamide were in common positions (Fig. 5).

AKH2, AKH3, AKH4, and ACP also exhibited a separation in the four first amino acids. In AKH2, there was a glycine residue after tryptophan, whereas AKH3, continues with a Glutamine; AKH4 and ACP from insects exhibited a glycine residue and another amino acid. Interestingly, all crustacean ACPs, including the one from *Callinectes toxotes*, exhibited two additional amino acids. The remaining AKH4 had a different organization before and after the tryptophan position, both in terms of number and amino acid sequence (Repository 6). The three amino acids after the tryptophan in the CRZ alignment exhibited the same pattern as AKH3, AKH4, and ACP. However, the most common repeat motif in insects and crustaceans, including *Callinectes arcuatus*, was Thr-Asn-Gly. Moreover, all CRZ exhibited Gln-Tyr as a repeat motif between phenylalanine and serine.

On the other hand, similar to the CRZ sequences, the GnRH sequence of most protostomes exhibited the same length before and after the tryptophan, sharing only the glycine at position one and serine at position five. Moreover, the GnRH of deuterostomes was two amino acids shorter and only shared the first glycine, the tryptophan, and the proline residues. Additionally, some GnRH isoforms from protostomes species exhibited the repeat motif Ser-Tyr-Gly > Leu.

### Phylogenetic Relationships Between the Neuropeptide Precursor Families

**Fig. 6:**
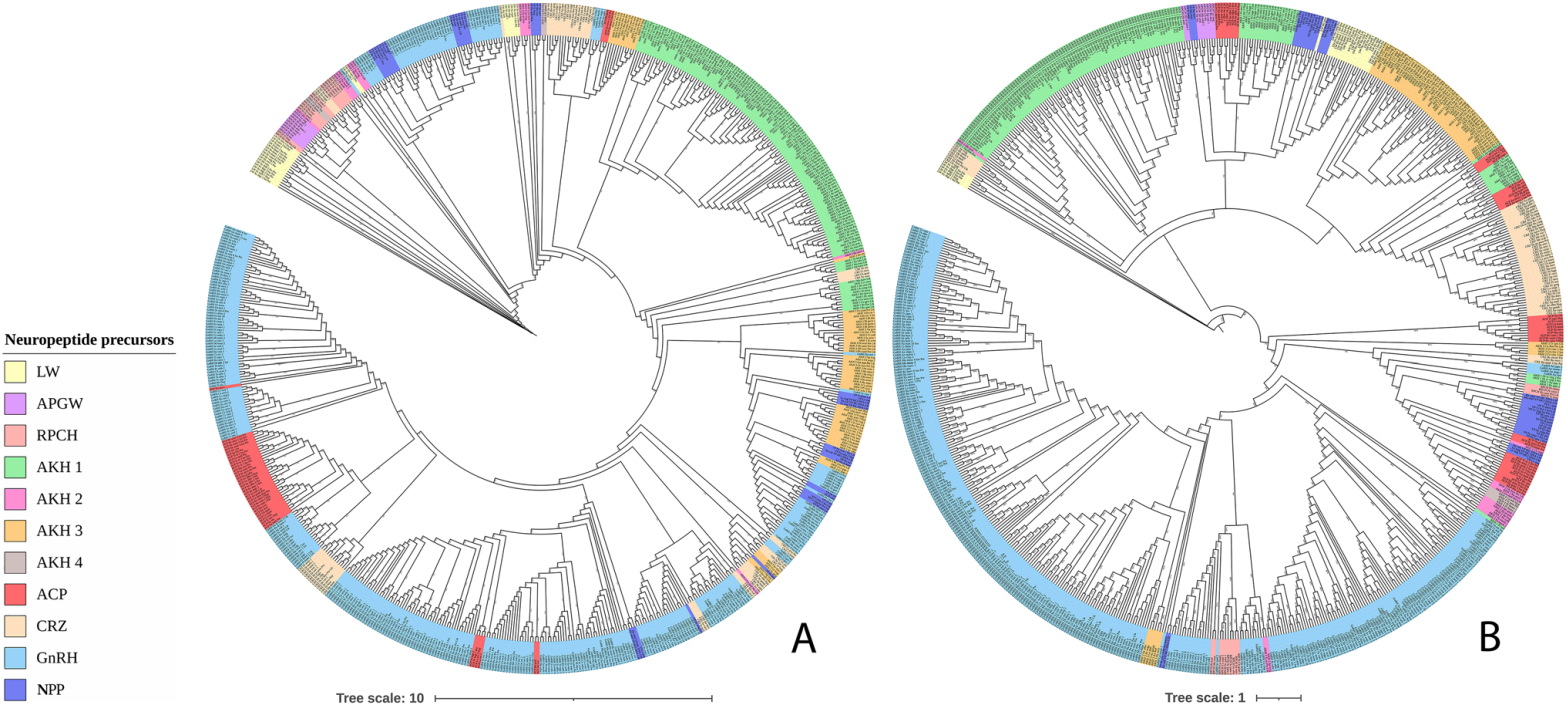
Phylogeny from all NPs grouped. Variations in the phylogenetic relationships between the precursor families when applying **A** the alignment parameters established by the DNA-LM and **B** the default parameters

The phylogeny generated with the DNA-LM parameters showed that the nodes and branches of vertebrate GnRH precursors were associated with other invertebrate neuropeptide precursors: thirty-nine of the forty-five for ACP, twenty-five of the fifty-three for CRZ, one of the thirteen AKH2, and eleven of the *NPPs*. Interestingly, forty-nine of the 323 GnRH precursors were determined in branches and nodes from other invertebrate precursors. Meanwhile, the Kalign parameters exhibited a clustering pattern according to the taxonomy of the species where they were identified. It is noteworthy that GnRH nodes corresponded to vertebrate species and showed other invertebrate precursors too. However, ten of the fourteen crustacean RPCH, a couple of the thirteen AKH2, six of the sixty-five AKH3, and two mollusks *NPP* showed branches associated with this family. The other neuropeptide families presented branches and nodes related to invertebrates (Fig. 6 and Repository 4).

Upon comparing the DNA-LM *versus* Kalign, the phylogenetic trees, where all neuropeptide precursors including APGW/RPCH *NPPs* and APGW/AKH *NPPs* were grouped, no similar results were identified between the trees. Additionally, taxon associations were not identified within each node. The roots in each tree represent the most ancient organisms from an evolutionary perspective, whereas the branches show the associations between more recent organisms. Therefore, the neuropeptide precursors and APGW/RPCH *NPPs* and APGW/AKH *NPPs* trees grouped with the DNA-LM parameters show stricter and more conserved associations among taxa (Fig. 6 and Repository 7).

**Fig. 7:**
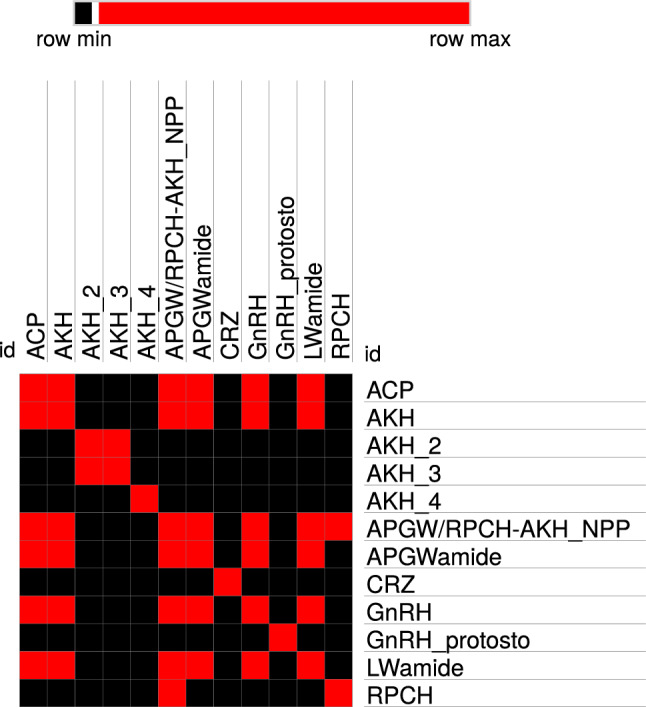
Orthogroup heat map for the neuropeptide families. The absence of correlation between the neuropeptide families is indicated in black (i.e., no orthogroups were formed between these families). The correlations between the families that formed possible orthogroups are shown in red (Color figure online)

The Robinson-Foulds metric showed a value of 19.74 between the tree generated with the Kalign software parameters and that of DNA-LM. Moreover, the homology relationship between the families showed no cleared orthogroups and therefore, a gene tree could not be obtained by OrthoFinder. There were four some possible orthogroups: one composed by LWamide, APGWamide, AKH1, ACP, GnRH, and APGW/RPCH-AKH *NPP*; another similar to this one but including RPCH; a third formed by AKH2 and AKH3; and a last one formed by RPCH and APGW/RPCH-AKH *NPP*. Moreover, AKH4, CRZ, and protostomes GnRH had individual group, and therefore, they did not had relation with previous orthogroups (Fig. 7 and Repository 7).

## Discussion

Molecular evolution and neuropeptide precursor origin have remained controversial throughout the twentieth and twenty-first centuries, particularly their participation in the evolution between protostomes and deuterostomes (King and Millar 1980; Sherwood and Parker 1990; Tsai 2006; Tsai and Zhang 2008; Derst et al. 2016; Semmens and Elphick 2017). Our study applied the DNA-LM to gain insights into the evolution of several neuropeptide families, including LWamide, APGWamide, RPCH, AKHs, ACP, CRZ, and GnRH, in addition to confirming the APGW/AKH *NPP*. Our findings demonstrated that a gene that codes for a neuropeptide precursor that contains AKH from insects, as well as the APGWamide copies from mollusks, were also present in the genome of the rotifer *B. plicatilis* (Blommaert et al. 2019) as it was predicted by the DNA-LM (Martínez-Pérez et al. 2007). Moreover, we employed an in silico approach to demonstrate the homology of the APGW/RPCH *NPP* and APGW/AKH *NPP* in mollusks, crustaceans, and insects, as well as the ACP and CRZ precursors in the transcriptomes of *Callinectes arcuatus* and *Callinectes toxotes* from the Gulf of California.

With the widespread adoption of NGS technologies, public sequence databases are constantly growing. NGS allows for the characterization of genomes and transcriptomes from model and non-model species, as well as from a wide range of ecosystems and non-described species (Levy and Myers 2016; Sayers et al. 2022). However, not all sequences reported as neuropeptide precursors possess the elements that identify them as such, including the signal peptide from the rough endoplasmic reticulum, the active peptides, related peptides, and excision motifs (Steiner et al. 1980; Rouillé et al. 1995; Kapp et al. 2009). Moreover, the relevant sequences have not been properly identified in several cases. Therefore, based on our findings, these elements must be considered basic structural requirements for a neuropeptide precursor to fulfill its cellular and physiological function. Additionally, the bioinformatic parameters for the assembly of the sequences obtained by NGS are sometimes inaccurate because a standard pattern has not been established, which highlights the need for the development of reliable and reproducible tools for the analysis of genomes or physiological processes in biomedicine, oncology, or dermatology (Kremer et al. 2005; Schuster 2008; Foulkes et al. 2017; Dotolo et al. 2022).

Consequently, inadequate sequence analysis generates biological misinterpretations and biases, resulting in questionable evolutionary interpretations. Therefore, new approaches are necessary for the storage and exchange of genomic and molecular data from neuropeptide precursors (Ekblom and Galindo 2011). Otherwise, the number of low-quality, mistagged, and/or mischaracterized neuropeptide precursor sequences will increase (Pible et al. 2014). In fact, the failure rates during the retrieval of neuropeptide precursor sequences are increasing, as reported in a previous study (Plachetzki et al. 2016). These problems could be overcome by using the BioDataToolKit and Proteios software followed by manual verification.

Only 5.21% of all available sequences in the GenBank database exhibited a correct neuropeptide precursor structure. The error increases when comparisons of newly isolated sequences and transcriptomes are made with respect to sequences or genomes that may have some of the previously described errors. Additionally, most studies have been conducted in model species (Steven et al. 2003; Chen et al. 2005; Lindemans et al. 2009; Sajwan et al. 2015), which has accelerated the generation of knowledge but leaves important information gaps. This knowledge gap can be filled by conducting comparative analyses including non-model species and wild organisms, as well as by conducting more rigorous studies. Using this approach, the ACP sequence from *Callinectes toxotes*, which until now was thought to be exclusively expressed in insects, was characterized (Hansen et al. 2010).

As reported by different authors (Gäde 1996; Tian et al. 2016; Sakai et al. 2017), some neuropeptide families are not exclusive to certain taxa as previously thought, which highlights the importance of in silico verification and the correlation of the taxonomic groups to which neuropeptides precursor families belong. For example, the GnRH family, which was previously thought to be exclusively expressed by members of the subphylum Vertebrata, is also present in the subphylum Tunicata, as well as the Echinodermata, Arthropoda, Mollusca, and Platyhelminthes phyla (Adams et al. 2003; Collins et al. 2010; Hasunuma and Terakado 2013; Semmens et al. 2016; Suwansa-ard et al. 2016). Similarly, CRZ was previously thought to be exclusive to insects but is now known to be also present in other arthropods (Nguyen et al. 2016) such as *Callinectes arcuatus*, as reported in this study.

The APGW/RPCH *NPP* and APGW/AKH *NPP* previously predicted by the DNA-LM exhibited homology with several expected species (Martínez-Pérez et al. 2007) but also with unexpected ones. At the time, the lack of accessibility to certain habitats, the low quality of sequencing methods, the intrinsic characteristics of reported sequences, and the low amount and diversity of species from which they came made it very difficult to validate the DNA-LM. However, the model has now been validated with the APGW/RPCH *NPP* of *A. californica* and *L. stagnalis* (Martínez-Pérez et al. 2007). The neuropeptide precursor APGW/RPCH *NPP* from these species is homologous with the protein BV898_10396 from the tardigrade *H. dujardini*, RPCHs from crustaceans, and some AKHs, whereas the sections corresponding to the APGW/RPCH *NPP* APGW/AKH *NPP* copies from APGWamide were homologous to the active peptide, the signal peptide, and the related peptide from APGWamide precursors from mollusks. This strongly suggests that the results derived from the DNA-LM correspond to an evolutionary mechanism through which new neuropeptide precursors are generated.

The phylogenetic trees of the grouped neuropeptide precursors APGW/RPCH *NPP* and APGW/AKH *NPP* generated with the Kalign and DNA-LM parameters exhibited a marked difference in the nodes for each family. This was consistent with previous findings (Tsai 2018) that identified a GnRH-like gene that could be classified as CRZ. In contrast, Plachetzki et al. 2016 proposed the existence of a superfamily composed of ACP, AKH, CRZ, and GnRH that could be classified as CRZ-like or AKH/CRZ-like. The orthology of these sequences has since been confirmed despite the nomenclature errors of some sequences (Tsai 2018). Our results strongly support both proposals. However, due to the results of our Robinson-Foulds metric analyses, we obtained a tree where GnRH and CRZ were clustered as two related superclades (Borozan et al. 2019). Similarly, there was not a clear identity homology relationship, because of the sequences variability, amino acids range, regions employed, the differences in evolutive relationships, and the lack of characterized amino acid sequences to some families. Moreover, algorithms employed by OrthoFinder are designed to generate sequences groups based on their common ancestry, which generated a partial result (Emms and Kelly 2015, 2019).

However, unlike in the aforementioned studies, the alignment made with the DNA-LM was based on codons that generate functional motifs from neuropeptide precursors, whereas the alignments made with the Kalign parameters are only based on amino acid similarities. The functional motifs of neuropeptide precursors have gradually changed for more than 500 million years since the Cambric explosion, as demonstrated in the alignment of all neuropeptide precursors. Importantly, constructing the phylogenetic trees using the DNA-LM parameters provides a novel means to identify these variations at the functional motif level between seemingly distinct neuropeptide precursors.

Interestingly, the alignment results and the phylogenies obtained with the DNA-LM and Kalign software were similar in the previously proposed GnRH division between protostomes and deuterostomes (Plachetzki et al. 2016; Tsai 2018). Nevertheless, the analyses conducted by these authors did not include the sequences of LWamide, APGWamide, RPCH, the four AKHs, and the APGW/RPCH *NPP*, which were presumably generated from the codon loss of the neuropeptide precursors LWamide and APGWamide. The alignments and phylogenies used herein were generated from the amino acid sequences from neuropeptide precursors. However, to confirm or propose any hypotheses or phylogenetic relationships between neuropeptide precursors in this study, the genes from each neuropeptide precursor family must be analyzed to corroborate the relationship between LWamide, APGWamide, RPCH, and AKH1, as originally proposed in the DNA-LM.

Therefore, the DNA-LM is needed to obtain the *NPPs* between LWamide/AKH3/CRZ/GnRH or different alternatives, thus allowing for the identification of codon loss in the regions and copies of the active peptide of LWamide or other neuropeptide precursors to determine the relationships between the GnRH of protostomes and deuterostomes. Additionally, neuropeptide precursors show a high degree of divergence in their amino acid sequences, and only small and highly conserved regions of certain genes such as active peptides or specific motifs within the peptide sequence present biological activity (Liu et al. 2006).

Finally, the APGW/RPCH *NPP* and APGW/AKH *NPP* shared homology with sequences from mollusks and arthropods, suggesting their presence in some undetermined or extinct species. The DNA-LM allows for the identification of phylogenetic relationships of amino acid functional domains among the LWamide, APGWamide, RPCH, AKHs, ACP, CRZ, and GnRH families of neuropeptides precursors. In this sense, the presence of APGW/AKH gene in *B. plicatilis* contributes to proposal that LWamide and GnRH could have been present in the common ancestor of the Eumetazoan (Jékely 2013), and therefore, DNA-LM may have been one of the evolutionary mechanisms explaining the diversity of current neuropeptides. However, the genes that encode for these neuropeptide precursors must be considered to establish the loss or gain of codons and confirm the evolutionary relationships among them.

## Data Availability

The datasets generated and/or analyzed during this study are publicly available on the Zenodo (Cadena-Caballero et al. 2022). https://doi.org/10.5281/zenodo.8092804.
